# The Living Fossil *Psilotum nudum* Has Cortical Fibers With Mannan-Based Cell Wall Matrix

**DOI:** 10.3389/fpls.2020.00488

**Published:** 2020-04-28

**Authors:** Tatyana Chernova, Marina Ageeva, Polina Mikshina, Oksana Trofimova, Liudmila Kozlova, Simcha Lev-Yadun, Tatyana Gorshkova

**Affiliations:** ^1^The Laboratory of Plant Cell Growth Mechanisms, Kazan Institute of Biochemistry and Biophysics, FRC Kazan Scientific Center of RAS, Kazan, Russia; ^2^Microscopy Cabinet, Kazan Institute of Biochemistry and Biophysics, FRC Kazan Scientific Center of RAS, Kazan, Russia; ^3^Laboratory of Plant Glycobiology, Kazan Institute of Biochemistry and Biophysics, FRC Kazan Scientific Center of RAS, Kazan, Russia; ^4^Department of Biology and Environment, Faculty of Natural Sciences, University of Haifa-Oranim, Tivon, Israel

**Keywords:** *Psilotum nudum*, plant fibers, cell wall, mannan, tracheids, immunocytochemistry

## Abstract

Cell wall thickening and development of secondary cell walls was a major step in plant terrestrialization that provided the mechanical support, effective functioning of water-conducting elements and fortification of the surface tissues. Despite its importance, the diversity, emergence and evolution of secondary cell walls in early land plants have been characterized quite poorly. Secondary cell walls can be present in different cell types with fibers being among the major ones. The necessity for mechanical support upon increasing plant height is widely recognized; however, identification of fibers in land plants of early taxa is quite limited. In an effort to partially fill this gap, we studied the fibers and the composition of cell walls in stems of the sporophyte of the living fossil *Psilotum nudum.* Various types of light microscopy, combined with partial tissue maceration demonstrated that this perennial, rootless, fern-like vascular plant, has abundant fibers located in the middle cortex. Extensive immunodetection of cell wall polymers together with various staining and monosaccharide analysis of cell wall constituents revealed that in *P. nudum*, the secondary cell wall of its cortical fibers is distinct from that of its tracheids. Primary cell walls of all tissues in *P*. *nudum* shoots are based on mannan, which is also common in other extant early land plants. Besides, the primary cell wall contains epitope for LM15 specific for xyloglucan and JIM7 that binds methylesterified homogalacturonans, two polymers common in the primary cell walls of higher plants. Xylan and lignin were detected as the major polymers in the secondary cell walls of *P*. *nudum* tracheids. However, the secondary cell wall in its cortical fibers is quite similar to their primary cell walls, i.e., enriched in mannan. The innermost secondary cell wall layer of its fibers but not its tracheids has epitope to bind the LM15, LM6, and LM5 antibodies recognizing, respectively, xyloglucan, arabinan and galactan. Together, our data provide the first description of a mannan-based cell wall in sclerenchyma fibers, and demonstrate in detail that the composition and structure of secondary cell wall in early land plants are not uniform in different tissues.

## Introduction

Plant fibers are sclerenchyma cells, mainly formed in order to participate in mechanical functions. They are elongated, functioning either dead or alive, and can have a single nucleus or many nuclei (Fahn, 1990). The major stages of plant fiber specialization are intrusive elongation and cell wall thickening (Gorshkova et al., 2012). Plant fibers are widely used as cordage and as raw material in the paper industry, and as a common part of many types of timber, they contribute to the mechanical properties of timber when used for construction, manufacturing furniture and other wooden items. Fibers also contribute significantly to the energy content of firewood, and are critical for reed and bamboo quality (e.g., Hill, 1952).

In higher plants, fibers can vary a lot in their cell wall composition and architecture (McDougall et al., 1993; Gorshkova et al., 2012; Sorieul et al., 2016). Distribution of cell wall polymers in fibers of angiosperms has been documented quite extensively (e.g., Blake et al., 2008; Bowling and Vaughn, 2008; Kim and Daniel, 2012; Guedes et al., 2017). All fibers of higher plants deposit secondary cell walls, which may contain several layers (S1, S2, S3) with different orientation of cellulose microfibrils. Cellulose, xylan and lignin in roughly equal proportions comprise the bulk of secondary cell walls in fibers of angiosperms (Harris, 2005; Gorshkova et al., 2012). The examples range from fibers in normal wood (Mellerowicz et al., 2001) to interfascicular and xylary fibers of *Arabidopsis* (Zhong et al., 2007). In addition to at least one layer of secondary cell wall, some fibers deposit a tertiary cell wall, also called G-layer, characterized by a high cellulose content, longitudinal orientation of its microfibrils, absence or low content of xylan and lignin, and rhamnogalacturonan I as a key non-cellulosic component (reviewed in Gorshkova et al., 2018). Deposition of tertiary cell walls can be constitutive, as in many fiber crops, or inducible, as in tension wood. Proportions of various layers in fibers developed in different species of angiosperms and in different growth conditions are quite variable, but the basic types of cell wall polymers in secondary and tertiary cell walls of higher plant fibers do not vary much, though there are nuances in structure.

The changes in fiber cell wall composition through evolution have barely been characterized. Thickened cell walls in early land plants were mainly studied in water-conducting cells (Friedman and Cook, 2000; Ligrone et al., 2002; Boyce et al., 2003; Carafa et al., 2005). Antibody-based screening of cell wall composition in ferns and lycophytes (Leroux et al., 2011, 2015) described thickened cell walls in sclerified and collenchymatous tissues of the cortex, but the definite cell types were not identified. These studies indicated that mechanical tissues in early land plants may be quite different from fibers of angiosperms. The specific architecture of the fiber cell wall, with axial orientation of cellulose microfibrils in the thick inner layer, was detected by Raman spectroscopy in *Equisetum hyemale* (Gierlinger et al., 2008). However, evolutionary aspects of fiber cell wall composition and structure have been discussed only with the emphasis on lignin distribution between primary and secondary cell walls in terms of the evolutionary derivation of both vessel elements and fibers from ancestral tracheids (Boyce et al., 2004). The limited information on the diversity and evolution of polysaccharide composition of fiber cell walls in early vascular land plants is partly due to the limited or lack of identification of sclerenchyma fibers in such taxa, and to the modes of fossilization.

We chose to study the constituents of the cell walls of cortical sclerified cells of the sporophyte of the living fossil *P. nudum* because of its uniqueness. This perennial rootless fern-like vascular plant, commonly known as whisk fern, usually grows as a small shrub and is found either as an epiphyte or growing in rocky habitats in tropical and subtropical regions all over the world (Gifford and Foster, 1989). *P. nudum* was once much cultivated in Japanese gardens as an ornamental plant. Over 100 garden varieties are known. Called matsubaran (“pine-needle orchid”) in Japanese, it was one of the noble plants in the Edo period (1603-1867). Valavan et al. (2016) reviewed numerous medicinal uses of whisk fern by local people in India and Hawaii, including wound healing.

While morphologically *P. nudum* sporophyte looks like the leafless Devonian early vascular plants (e.g., Gifford and Foster, 1989), molecular studies have shown that it is closely related to *Equisetum*, and it is more advanced than *Selaginella*, *Isoetes*, and *Lycopodium* (Ruhfel et al., 2014). While members of the genus *Psilotum* appear as if belonging to a much older leafless tracheophyte group from the Rhynie chert rather than to the current ferns, their unique morphology seems to be secondary, representing the reduction of characters rather than being even more ancestral than their actual quite basal position (Ruhfel et al., 2014). Thickened secondary cell walls in *P*. *nudum* have previously been analyzed mainly for stele components (Carafa et al., 2005; Leroux et al., 2015) and stomata cells (Carafa et al., 2005), while there are also sclerified cells in the middle cortex (Ford, 1904; Vahdati et al., 2014). We demonstrate that these cortical cells are fibers and that they have secondary cell walls different from the cell walls found in *P. nudum* tracheids and from fibers of higher plants.

## Materials and Methods

### Plant Material

Plants of various sizes and ages of *P. nudum* were obtained from the Tropical Greenhouse of the Botanical Gardens of Tel Aviv University, Tel Aviv, Israel. Stems and branches of several orders from five individual plants were sampled and used for cell wall studies. The major stem was determined as the first branching order, the branches just above the first bifurcation were the second order, and so on.

In an immunochemical study, we used for comparison with *P. nudum* the angiosperm flax (*Linum usitatissimum* L., cultivar Mogilevsky from the collection of the All-Russian Flax Research Institute, Torzhok). Plants were grown under open air in boxes with a 50 cm soil layer and received natural daylight and daily watering. Samples for analysis were collected at the fast-growth period of plant development when the stem height was 25-28 cm (30 days after sowing).

### Light Microscopy

Middle parts of stems and branches of various branching orders of *P. nudum* were fixed in Clarke’s solution (ethanol + acetic acid - 3:1). Longitudinal and transverse sections were made using vibratome VT1000S (Leica Biosystems, Germany) or by hand using a razor blade. Sections were stained for the presence of lignin with 0.5% (w/v) toluidine blue in water, or with phloroglucinol-HCl (Jensen, 1962). To better visualize cell walls, tissues were stained with 0.003% (w/v) Calcofluor White (Megazyme, Wicklow, Ireland) in Tris-buffer pH 7.2. For nuclei visualization, isolated fibers were stained with DAPI (4′, 6-diamidino-2-phenylindole) in a concentration of 1 μg/mL in water. For starch detection, the sections were stained in 2% alcohol iodine solution for 20 s. All specimens were studied under a LSM 510 Meta confocal microscope (Zeiss, Germany) with UV excitation from a HBO mercury vapor lamp (Zeiss, Germany) for Calcofluor White and DAPI fluorescence, or using the transmitted light for toluidine blue, iodine and phloroglucinol staining, and photographed with an AxioCam HRs camera (Zeiss, Germany). For UV light, a 365 nm bandpass excitation filter, a 395 nm color splitter, and a 397 nm long pass filter (FSET01) from a HBO mercury vapor lamp (Zeiss, Germany) were used. Microscopy under polarized light was performed on an Axio microscope (Zeiss, Germany) equipped with a polarizing filter.

### Tissue Maceration

Individual *P. nudum* fibers were isolated by maceration after tissue incubation in 3% (w/v) Macerozyme (Serva, Heidelberg, Germany) in 100 mM phosphate-buffered saline (PBS) (pH 6.0) overnight at 30°C. After washing in the same buffer, parts of the stems were transferred to glass slides, where fibers were separated from the surrounding tissues with the aid of a needle.

### Immunocytochemistry

For immunocytochemistry analysis of the cell walls, 5 mm long samples from the middle part of third order of *P*. *nudum* shoot and from the middle part of *L*. *usitatissimum* stem were fixed according to a standard procedure in a mixture of 3% (w/v) paraformaldehyde and 0.5% (v/v) glutaraldehyde in 0.1 M phosphate buffer (pH 7.2) for 4 h at room temperature. Then, the samples were dehydrated in a graded aqueous ethanol series and acetone, immersed in LR White resin (Ted Pella, Inc., California) that contained acetone in the proportions (v/v) 1:4, 2:3, 3:2, 4:1 with each step involving a 12–24 h incubation. The samples were then embedded in pure LR White resin in Beem capsules and polymerized at 60°C for 24 h. Semi-thin transverse sections (1 μm thick) were prepared using a glass knife on a LKB 8800 ultramicrotome (LKB Instruments, Stockholm, Sweden) and collected on silane-coated microscope slides. To compare the distribution of epitopes for LM11 and LM21 antibodies in various orders of *P*. *nudum* shoots, an additional set of transverse sections (50 μm thick) was prepared using a Leica VT1000S vibratome (Leica Biosystems, Germany).

The immunohistochemical detection was performed using the antibodies: LM5, LM6, LM11, LM15, LM21, JIM5, JIM7, JIM14, and BG1 (Table 1). Sections were incubated in Na-phosphate buffered saline (PBS), pH 7.4, containing 3% (w/v) bovine serum albumin (BSA) for 1 h to block non-specific labeling. Then the sections were incubated for 1 h with primary antibodies diluted 1:10. The secondary antibodies – goat anti-mouse for BG1, and goat anti-rat antibodies for others – linked to fluorescein isothiocyanate (488 nm) (FITC; Sigma) were used in a dilution 1:100 for 1 h in darkness. The sections were mounted in CFM-1 mounting solution (Electron Microscopic Sciences, Hatfield, PA, United States). All antibodies were diluted in 0.1 M PBS containing 0.06% (w/v) BSA. All incubations were performed at room temperature. Primary antibodies were omitted in control experiments. Cross-sections of maize roots were used as a positive control for the BG1 antibody, and flax stem sections were used as a positive control for the JIM14 antibodies.

**TABLE 1:** Distribution of cell wall epitopes for antibodies specific for various polymers in *P. nudum* stem tissues.

Antibody/references	Antigen/epitope	Tissue of *P. nudum* stem								
		
		Epidermis	Outer cortex	Middle cortex		Inner cortex	Endodermis	Phloem	Xylem	
								
				ML + PCW	SCW				ML + PCW	SCW
LM21 (Marcus et al., 2010)	(galacto) (gluco)mannan	+++	+++	+++	+++	+++	±^1^	+^1^	±	±
LM11 (McCartney et al., 2005)	(1→4)-β-D-xylan/arabinoxylan	+^2^	−	−	+++	−	−	−	−	+++
LM15 (Marcus et al., 2008)	XXXG motif of xyloglucan	+++	+++	+++	++^3^	+	+	+++	±	±
JIM5 (Knox et al., 1990)	Partially Me-HG/de-esterified HG	++	++	−	±	−	−	−	−	−
JIM7 (Knox et al., 1990)	Partially Me-HG	+++	+++	++	+	+	+	+++	+	−
LM5 (Jones et al., 1997)	(1→4)-β-D-galactan	++	++	−	+++^3^	+	−	−	−	+++
LM6 (Willats et al., 1998)	(1→5)-α-L-arabinan	+^2^	−	−	++^3^	±	−	−	−	−
JIM14* (Knox et al., 1991)	Arabinogalactan protein	−	−	−	−	−	−	−	−	−
BG1* (Meikle et al., 1994)	(1→3)-(1→4)-β-D-glucan	−	−	−	−	−	−	−	−	−

*+++ very strong labeling; ++ strong labeling; + weak labeling; ± very weak and scattered labeling; – no labeling; ^1^restricted to primary cell wall and cell corners; ^2^restricted to stomata cell; ^3^restricted to the innermost cell wall region. ^∗^JIM14 and BG1 did not bind to any tissue in *P. nudum* stem but worked well on the positive controls.*

The sections were examined using a laser confocal fluorescence microscope (LSM 510 Meta; Carl Zeiss, Jena, Germany). Immunofluorescence was observed by using excitation at 488 nm and emission at 503–550 nm. The transmitted light channel was used for the detection of anatomical details. Fluorescence detection settings (laser intensities, pinhole sizes, gain settings, offset) used to compare stem sections at the same magnification, were kept at the same level for all antibodies.

### Monosaccharide Composition of Alcohol-Insoluble Residue (AIR) of *P. nudum* Shoots

The *P. nudum* shoots of first, second and fourth orders were fixed like the samples used for light microscopy. Then they were sponged up from the fixator solution by a filter paper and weighed. The material was homogenized in liquid nitrogen and washed into Eppendorf tubes by 2 mL of 96% ethanol. The homogenate was clarified by centrifugation at 10,000 × *g* for 10 min. The supernatant was removed and samples were washed three times by 96% ethanol, lyophilized and weighted. The dried material was considered as alcohol-insoluble residue (AIR). Two milligrams of AIR were placed into Pyrex tubes, and 500 μL of 2 M TFA were added. Hydrolysis was carried out at 120°C for 1 h. The material was then dried at 60°C with the flow of air. Samples were dissolved in MilliQ water and analyzed by high-performance anion-exchange chromatography using a DX-500 system (Dionex, United States) equipped with a CarboPac PA20 guard column (3 × 30 mm, Thermo, United States) and CarboPac PA20 analytical column (3 × 150 mm, Thermo, United States). For the analysis of monosaccharides, pulsed amperometric detection with waveform A that uses negative potentials for electrode cleaning and provides the reproducibility of electrochemical response for a long time was applied. The column’s temperature was 30°C, and the mobile phase was pumped at 0.5 mL.min^–1^. Eluents: A – 200 mM NaOH, B–100 mM NaOH in 1 M NaOAc, and C–MilliQ water. Neutral sugar composition was determined at isocratic separation by 4 mM NaOH [A/C – 2/98 (%)] during 15 min that allowed xylose and mannose to be well resolved. Before the analysis of the next sample the column was washed by 200 mM NaOH (A – 100%) for 10 min and equilibrated by 4 mM NaOH (A/C – 2/98 (%)] at 35 min. Uronic acid content was analyzed on the same samples at the following elution scheme: 0–10 min A/C– 2/98 (%); separation of charged carbohydrates – 10–11 min linear gradient until A/B/C – 6.8/10/83.2 (%); 11–16 min linear gradient until A/B/C – 5.3/30/64.7 (%); 16–17 min linear gradient until A – 100%; cleaning and equilibration of column – 17–30 min A – 100%; 30–31 min linear gradient until A/C – 2/98 (%); 31–60 min A/C – 2/98 (%). The results were analyzed using the PeakNet 4.30 software according to the calibrations obtained for monosaccharide standards treated in advance with 2M TFA at 120°C for 1 h. The analysis was performed for four independent biological replicates and two analytical replicates. Results from this analysis in diagrams are conveyed as means ± SD.

## Results

### Cortical Fibers Are Present in *P. nudum* Stem

**FIGURE 1:**
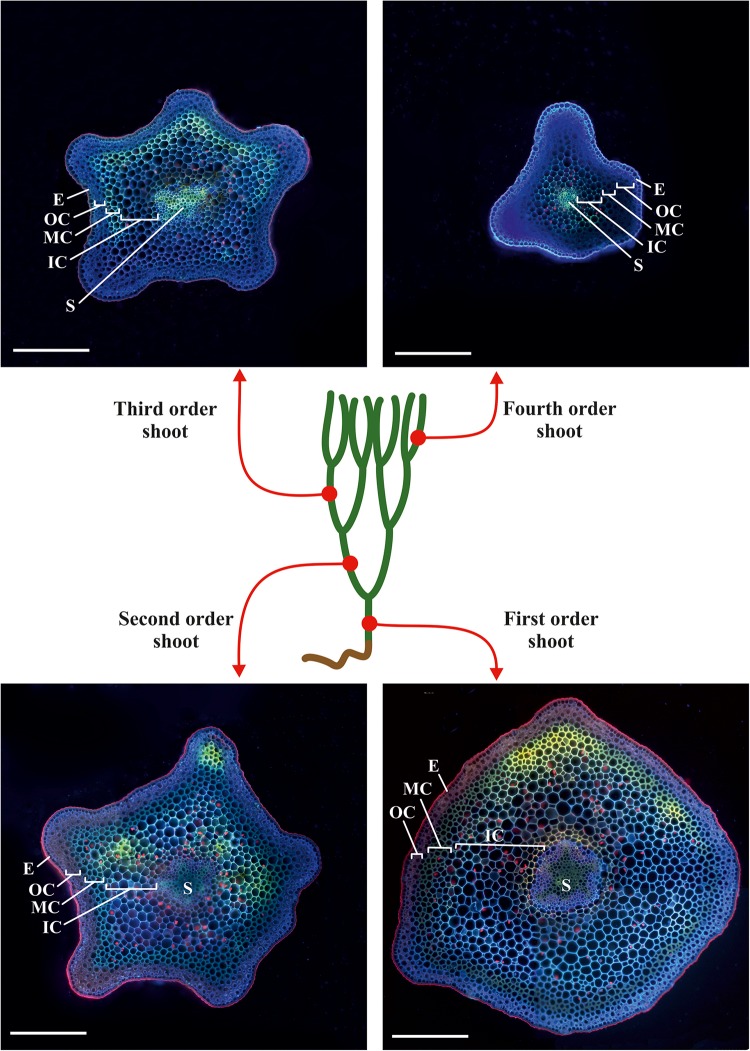
Scheme of sampling sites on a typical plant of *P. nudum* for cross-sections of different order shoots. The cross-sections were stained with Calcofluor White and photographed under ultraviolet light. E – epidermis, IC – inner cortex, MC – middle cortex, OC – outer cortex, S – stele. Scale bar 500 μm.

**FIGURE 2:**
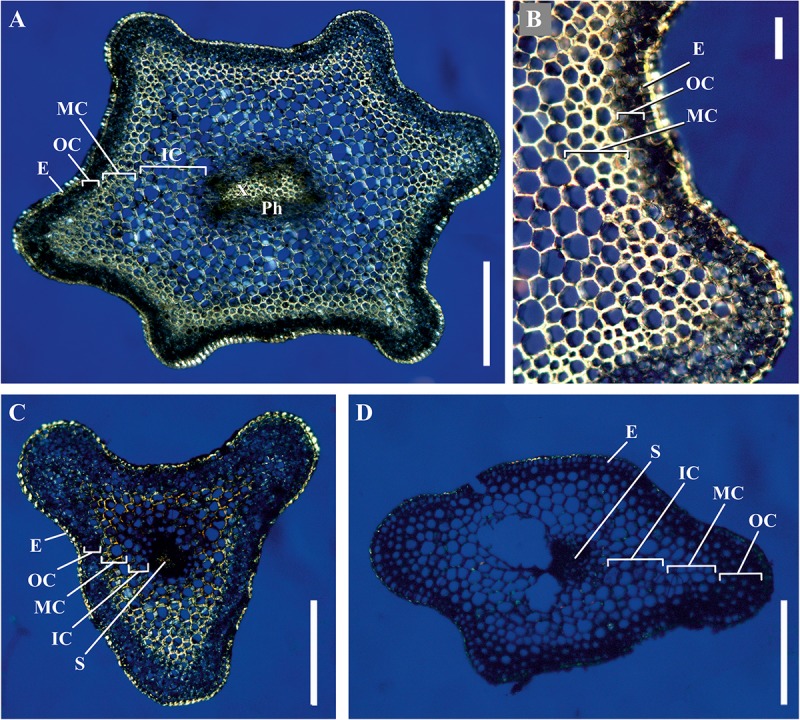
Cross-sections of *P. nudum* shoots under polarized light. **(A,B)** First order shoot, **(C)** third, and **(D)** fifth order shoots. Illuminated birefringent ring of fibers is present in the first- and third order shoots and absent in the fifth order shoot. E – epidermis, IC – inner cortex, MC – middle cortex, OC – outer cortex, Ph – phloem, S – stele, X – xylem. Scale bar **(A,C,D)** 500 μm, **(B)** 100 μm.

We analyzed the general plant anatomy of the *P. nudum* stems on cross-sections of the several shoot orders (Figure 1), which was in accordance with previous studies (Ford, 1904; Vahdati et al., 2014). Together with epidermis and stele, there were three layers of cortex in the *P. nudum* shoot: the outer cortex consisting of 2–4 layers of chlorophyllous cells, the middle cortex comprising 2–5 layers of sclerenchymatous cells, and the inner cortex, including thin-walled parenchymatous cells. Polarized light helped to visualize the thick walls in tracheids, epidermis and numerous cells located in the middle cortex (Figure 2). Cells with thickened cell walls were more pronounced in the cross-sections of lower order *P. nudum* stems, and were barely present in the younger (fifth) order stem segments (Figures 1, 2). The middle cortex varied in number of cell layers (depending on position) in some stem sections (Figure 2C).

**FIGURE 3:**
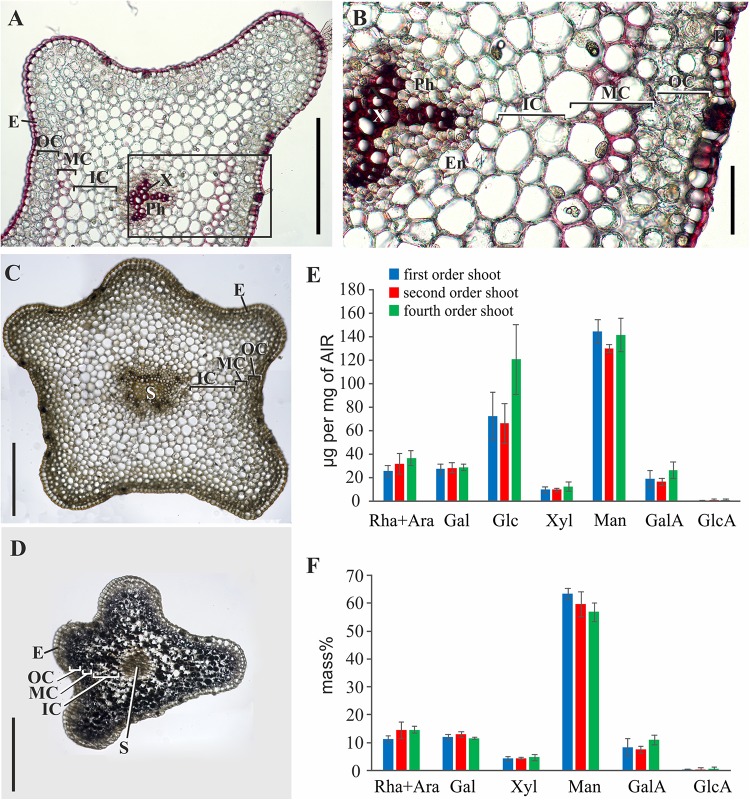
Characterization of polysaccharides and cell wall phenolic compounds in *P. nudum* stem. **(A)** Cross-sections of second order shoot stained by phloroglucinol-HCl (thickened cell walls are stained in red). **(B)** An enlarged image of the area indicated by a square in **(A)**. **(C,D)** Staining with iodine to reveal starch in **(C)** first order and **(D)** fourth order shoots.) **(E,F)** Monosaccharide composition of alcohol-insoluble residue in shoots of several orders: **(E)** Yield of individual monosaccharides (μg/mg of AIR) and **(F)** mass % of monosaccharides (without Glc) released after TFA-hydrolysis. En – endodermis, E – epidermis, IC – inner cortex, MC – middle cortex, OC – outer cortex, Ph – phloem, S – stele, X – xylem. Scale bar **(A,C,D)** 500 μm, **(B)** 100 μm.

Phloroglucinol-HCl staining of stem cross-sections indicated that thickened cell walls contained phenolic components; the intensity of staining was considerably higher in tracheids than in the middle cortex cells (Figures 3A,B). In the latter, staining with phloroglucinol-HCl was rather uneven (Figure 3). The iodine test revealed a high starch content in younger shoots (Figures 3C,D). This coincided with the higher yield of glucose among monosaccharides released by TFA-hydrolysis of polysaccharides from the alcohol-insoluble residue of younger shoot samples (fourth order) (Figure 3E). The rest of monosaccharides originated from cell wall polymers; their composition with a high prevalence of mannose was similar in shoots of different orders (Figure 3F). However, the yield of alcohol-insoluble residue (AIR) in older order shoots of *P*. *nudum* stem was considerably higher, rising from 7 ± 1% in the fourth order to 20 ± 2% in the first order and reflecting either cell wall thickening or decrease of water content.

**FIGURE 4:**
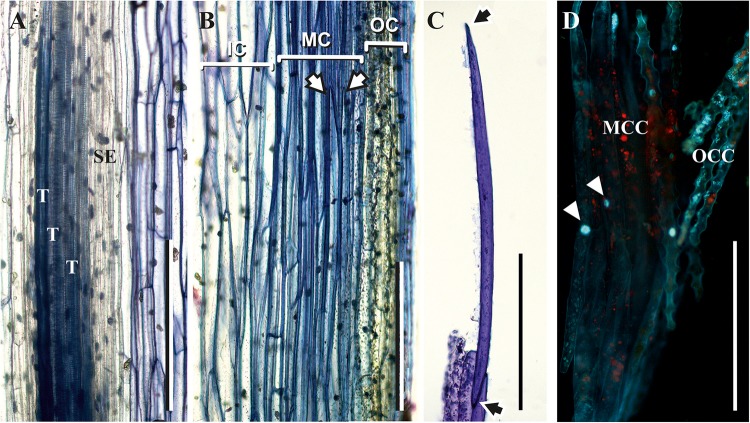
Fibers in the middle cortex of *P. nudum.* **(A,B)** Longitudinal sections of the first order shoot from stele and cortex, correspondingly, stained with 0.5% toluidine blue; **(C)** elongated cells with pointed ends (marked by arrows) isolated from cortex tissues of the first order shoot by maceration and stained with 0.5% toluidine blue; **(D)** partially macerated cortex tissues stained with DAPI (nuclei in elongated cells of middle cortex are marked by arrowheads). E – epidermis, IC – inner cortex, MC – middle cortex, MCC – middle cortex isolated cells, OC – outer cortex, OCC – outer cortex isolated cells, T – tracheid, SE – sieve element. Scale bar 500 μm.

The thick-walled cortical cells had an elongated shape and pointed ends, as seen in the longitudinal stem sections (Figure 4B) and in cells isolated from cortical tissues by maceration (Figure 4C). The average length of such cells was close to 1,000 μm, and their diameter was about 40 μm. According to their morphology, these cortical cells are typical sclerenchyma fibers. Staining of partially macerated cortex tissues with DAPI showed only one nucleus in the elongated cells of the middle cortex (Figure 4D). Longitudinal sections did not reveal fibers in the stele region, which contained only tracheids and thin-walled cells (Figure 4A).

### The Existence of a Thick Layer of Xylan in Mannan-Based Cell Walls of *P. nudum* Fibers Is Revealed by Immunocytochemistry

**FIGURE 5:**
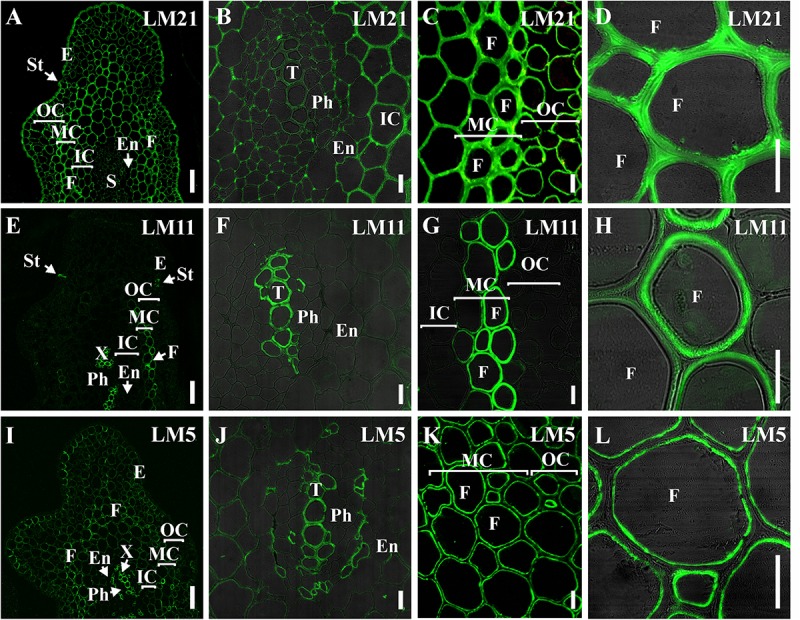
Immunolabeling of cross-sections of *P. nudum* third order shoot with **(A–D)** LM21 monoclonal antibody specific for mannan, **(E–H)** LM11 monoclonal antibody specific for β-(1,4)-xylan/arabinoxylan and **(I–L)** LM5 monoclonal antibody specific for β-(1,4)-D-galactan. **(A,E,I)** General view of the part of shoot, **(B,F,J)** stele area, **(C,G,K)** cortex cells, **(D,H,L)** fibers of middle cortex. **(D,F,H,L)** Fluorescent signal superimposed on the transmitted light image. References for probe specificity are listed in Table 1. En – endodermis, E – epidermis, F – fibers, IC – inner cortex, MC – middle cortex, OC – outer cortex, Ph – phloem, S – stele, St – stomata cells, T – tracheid, X – xylem. Scale bar **(A,E,I)** 100 μm, **(B–D,F–H,J–L)** 20 μm.

**FIGURE 6:**
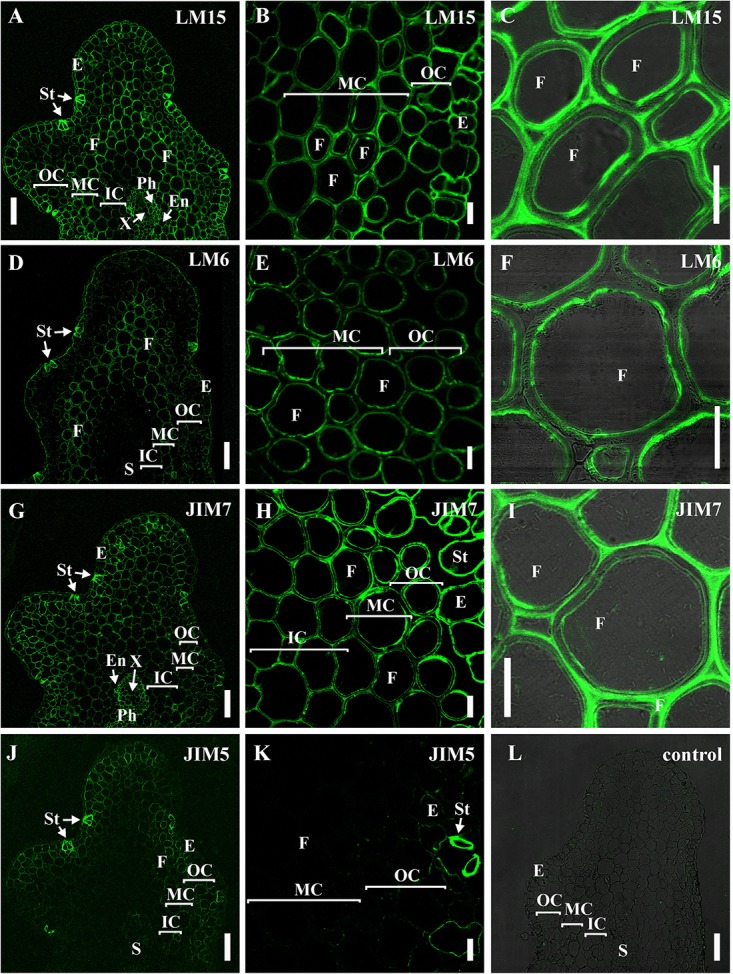
Immunolabeling of cross-sections of *P. nudum* third order shoot with **(A–C)** LM15 monoclonal antibody specific for xyloglucan, **(D–F)** LM6 monoclonal antibody specific for α-(1,5)-L-arabinan, **(G–I)** JIM7 and **(J,K)** monoclonal antibodies specific for homogalacturonans with different degree of methylesterification. **(A,D,G,J)** General view of the part of shoot, **(B,E,H,K)** cortex cells, **(C,F,I)** fibers of middle cortex. **(C,F,I)** Fluorescent signal superimposed on the transmitted light image. **(L)** Control with the omitted primary antibody. References for probe specificity are listed in Table 1. En – endodermis, E – epidermis, F – fibers, IC – inner cortex, MC – middle cortex, OC – outer cortex, Ph – phloem, S – stele, St – stomata cells, X – xylem. Scale bar **(A,D,G,J)** 100 μm, **(B,C,E,F,H,I,K)** 20 μm.

The presence and distribution of cell wall polysaccharides in thick-walled cells of *P. nudum* stems were analyzed by using various antibodies specific to several cell wall polysaccharides (Table 1, Figures 5, 6). The epitope of the antibody recognizing mannan (LM21) was found in cell walls of all the cells of various stem tissues, with their highest level of detection in the middle cortex fibers (Figures 5A–D). LM21 epitope was located throughout the entire thickness of fiber cell walls, including the material of intercellular spaces and outer cell wall layers (Figure 5D). Only a thin outer cell wall layer was labeled by LM21 in tracheids (Figure 5B).

The epitope of the LM11 antibody specific to β-(1,4)-xylan/arabinoxylan (Figures 5E–H) was found in thickened cell walls of sclerenchyma fibers, in addition to the cell walls of tracheids and stomata cells that were described in an earlier study (Carafa et al., 2005). However, not all the fibers located along the perimeter of the same stem cross-section had epitopes to this antibody in their cell wall (Figure 5H). When LM11 antibody epitope was present in the fiber cell wall, they were distributed only throughout the thickened secondary cell wall layer and neither in the primary cell walls nor in the middle lamella (Figures 5G,H), the same pattern as in tracheids (Figure 5F).

The antibody specific to β-(1,4)-D-galactan (LM5) bound to the cell walls of many tissues in stems of *P*. *nudum*, but with different distribution between cell wall layers (Table 1, Figures 5I–L). In fibers with a thickened secondary cell wall, it was especially evident that epitope of LM5 antibody was located only along the innermost layer of the secondary cell wall (Figure 5L). In contrast, LM5 labeled the whole thickness of the secondary cell wall in tracheids (Figure 5J).

The innermost portion of thickened cell wall in fibers of *P*. *nudum* also had epitope for the LM15 specific to xyloglucan (Figure 6C) and for LM6 raised to bind α-(1,5)-L-arabinan (Figure 6F). In fibers, LM15 was also bound to the primary cell walls as distinct from LM6. In the xylem, labeling of the secondary cell wall’s innermost layer similar to that in fibers was never detected (Table 1). LM6 and JIM5 heavily labeled stomata cell pairs in the epidermis (Figures 6D,K), as described earlier for this cell type in various plant species, including early land plants (Merced and Renzaglia, 2014, 2019). The epitopes of the antibodies specific to mixed-linkage glucan (BG1) and to the arabinogalactan protein (JIM 14), were not revealed in the cell walls of any tissue of the *P. nudum* stem, including the fibers (Table 1).

The epitopes of the antibody specific to partially methylated homogalacturonan (JIM7), or the antibody specific to partially methylated and de-esterified homogalacturonan (JIM5), did not have the same location in stem tissues (Figures 6G–K). The epitope of the JIM5 antibody was distributed in the cell walls of the epidermis and the outer cortex cells (Figures 6J,K). The epitope of JIM7 was located in the cell walls of all stem tissue cells (Figures 6G–I), especially in the epidermis, in cells of the outer and middle cortex, and in the phloem.

**FIGURE 7:**
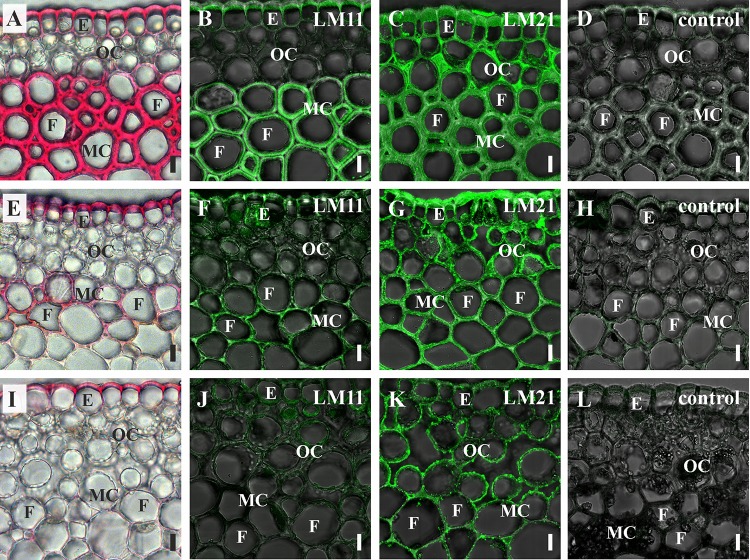
Cross-sections of *P. nudum* shoots of different order. **(A–D)** First order shoot, **(E–H)** third order shoot, and **(I–L)** fifth order shoot. **(A,E,I)** Stained by phloroglucinol-HCl (thickened cell walls are stained in red). Immunolabeling with **(B,F,J)** LM11 monoclonal antibody specific for β-(1,4)-xylan/arabinoxylan and **(C,G,K)** LM21 monoclonal antibody specific for mannan. **(D,H,L)** Controls with the omitted primary antibody. **(B–D,F–H,J–L)** Fluorescent signal superimposed on the transmitted light image. References for probe specificity are listed in Table 1. E – epidermis, F – fibers, MC – middle cortex, OC – outer cortex. Scale bar 20 μm.

Binding of LM11 by fiber cell walls went in parallel with phloroglucinol-HCl staining, as evidenced by comparison of several shoot orders (Figure 7). Cortical cells of the younger shoot order, all of which had only primary cell walls with abundant epitope for LM21, did not contain epitope for LM11 and were not stained with phloroglucinol-HCl. Both binding of LM11 and staining with phloroglucinol-HCl were detected in the middle shoot order, being rather uneven (Figures 3, 7D,E). In the lowest shoot order, staining of cortical fibers was the most intensive, the same as labeling with LM11 and LM21. However, only secondary cell walls bound the anti-xylan antibody (Figure 7B), while phloroglucinol-HCl stained all layers of fiber cell walls (Figure 7A). Labeling by anti-mannan antibody occurred throughout the entire cell wall in fibers of all shoot orders (Figures 7C,F,I).

**FIGURE 8:**
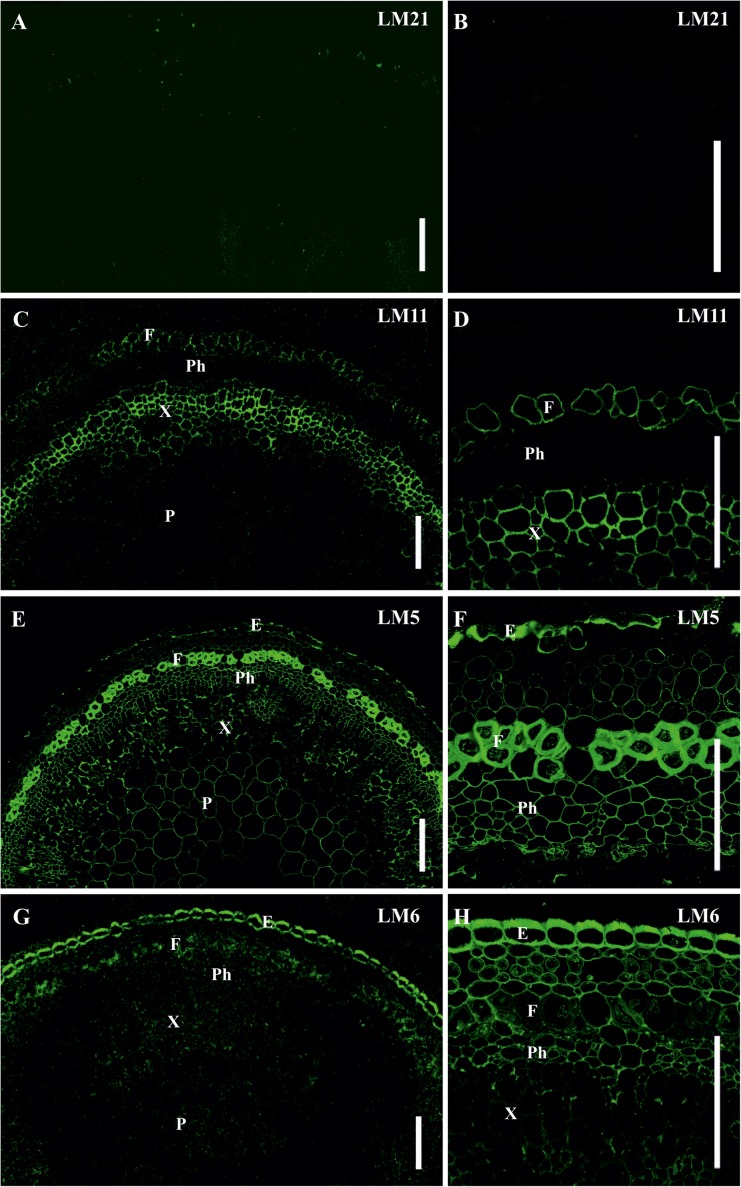
Immunolabeling of cross-sections of *L. usitatissimum* stem with **(A,B)** LM21 monoclonal antibody specific for mannan, **(C,D)** LM11 monoclonal antibody specific for β-(1,4)-xylan/arabinoxylan, **(E,F)** LM5 monoclonal antibody specific for β-(1,4)-D-galactan and **(G,H)** LM6 monoclonal antibody specific for α-(1,5)-L-arabinan. **(A,C,E,G)** General view of the part of stem, **(B,D,F,H)** cortex and phloem cells. E – epidermis, F – fibers, P – parenchyma, Ph – phloem, X – xylem. Scale bar 100 μm.

In order to compare the distribution pattern of the epitopes for the antibodies used in *P*. *nudum* with a higher plant, we used stems of flax (*L. usitatissimum*), which is renowned for its fibers and has typical angiosperm cell types with thickened cell walls (Figure 8). Xylem tissues of flax (including xylary fibers) develop secondary cell walls; primary phloem fibers of flax deposit thick tertiary cell walls in addition to thin primary and secondary cell walls (Gorshkova et al., 2018). In flax, both major types of fibers present in angiosperms – with xylan-based secondary cell walls and with highly cellulosic cell walls – can be characterized on the same stem cross section.

The epitope for mannan-recognizing LM21 antibody was hardly detected in flax stem tissues (Figures 8A,B). LM11 antibody specific for β-(1,4)-xylan/arabinoxylan bound the secondary cell walls in xylem cells and the thin layer in phloem fibers (Figures 8C,D). The epitope of LM5 antibody that recognizes β-(1,4)-galactans was abundant in the tertiary cell walls of phloem fibers, were present in the primary cell walls of many tissues and in some parts of the thickened cell walls in the epidermis, but were not detected in the secondary cell walls of the xylem (Figures 8E,F). LM6 antibody specific for α-(1,5)-L-arabinan labeled the primary cell walls in many tissues and the thickened cell wall of epidermal cells (Figures 8G,H).

## Discussion

### Primary Cell Walls in all Tissues of *P. nudum* Stem Are Mannan-Enriched

Primary cell walls of early land plants are known to be enriched in (gluco)mannan. Based on the research of cell wall composition in young shoots of several bryophytes, lycophytes and ferns, mannans were described as the principal cross-linking glycans in their primary cell walls (Popper and Fry, 2003, 2004; Silva et al., 2011). A proposal has been made to refer the mannan-rich primary cell walls of these taxonomic groups as fundamentally distinct among land plants, the Type III primary cell wall, different from the earlier identified types I and II, characteristic for dicots and grasses, respectively (Silva et al., 2011). In our study, primary cell walls in all stem tissues of *P*. *nudum* were labeled by the LM21 antibody specific for (gluco)mannan (Figures 5A–D), while in flax the epitope for LM21 was hardly detected (Figures 7A,B). In various orders of *P*. *nudum* stem, mannose was the major constituent of the TFA-hydrolysable polysaccharides (Figures 3E,F). Primary cell walls of all tissues in *P*. *nudum* shoots also contain xyloglucan and methylesterified homogalacuronan, as evidenced by labeling with the antibodies LM15 and JIM7, respectively (Figures 6A–C,G–I). This is in accordance with previous studies (Popper and Fry, 2004; Leroux et al., 2015).

The primary cell wall of cortical fibers is, altogether, quite similar to the surrounding tissues in the capacity to bind various antibodies (Figures 5, 6). The most pronounced binding is detected for LM21 (Figures 5A–D). The epitope of JIM7 is more abundant in cortical fibers than in inner cortex cells, indicating higher content of methylesterified homogalacuronan in primary cell walls of fibers (Figures 6G–I).

### The Secondary Cell Wall in Cortical Fibers of *P. nudum* Differs From That of Tracheids

*Psilotum nudum* is one of the earliest extant vascular plant taxa that has water-conducting tissues with secondary cell walls similar to those of angiosperms, i.e., containing, in addition to cellulose, β-(1,4)-D-xylan and lignin. Water-conducting cells with secondary walls reinforced by lignin are considered as the basic feature of Tracheophyta (Kenrick and Crane, 1997). The earlier taxonomic vascular taxa do not have xylans, or have xylans with different types of linkages, such as β-(1,3)-xylans (Harris, 2005; Sørensen et al., 2011; Smith et al., 2017; Hsieh and Harris, 2019), or mixed-linkage xylans (Viana et al., 2011), or have β-(1,4)-D-xylan in cell types other than tracheids, e.g., leaf cells and axillary hairs in *Physcomitrella patens* (Kulkarni et al., 2012). The first thickened cell walls of water-conducting elements, such as moss hydroids, are non-lignified (Boyce et al., 2003). Immunocytochemical studies of the thickened cell wall areas in the hydroids of polytrichopsid mosses also suggest that they are not secondary cell walls (Ligrone et al., 2002; Carafa et al., 2005).

In *P*. *nudum*, the presence of β-(1,4)-xylan in cell walls in tracheids as well as in Casparian strips in endodermal cells and in stomata cells was detected earlier by labeling with the antibodies LM10 and LM11, which recognize this polymer (Carafa et al., 2005). Electron microscopy confirmed antibody binding to the secondary cell walls and the absence of binding to adjacent primary cell walls. As for lignin presence, K. Boyce with colleagues used tracheids of *P*. *nudum* stems as an extant reference to detect lignin in Lower Devonian plant fossils (Boyce et al., 2003). Lignin distribution is also illustrated by tissue staining with phloroglucinol-HCl (Figures 3, 7). The combination of β-(1,4)-xylan and lignin in thickened cell walls of *P*. *nudum* tracheids allows to consider it to be quite similar to the secondary cell walls of xylem tissues in angiosperms (e.g., Figures 8C,D). The major peculiarity of the secondary cell wall in tracheids of *P*. *nudum* that distinguishes it both from the secondary cell wall in cortical fibers of the same plant and from the secondary cell walls of xylem tissues in angiosperms, is the presence of β-(1,4)-galactans detected by LM5 antibody (Figure 5J). Such polymers (with specific nuances in structure) are present in compression wood of gymnosperms (Chavan et al., 2015) and in tertiary cell walls of many plant fibers of dicots (Gorshkova et al., 2018; Figures 8E,F).

The stele of *P*. *nudum* does not contain fibers, as seen on the longitudinal sections (Figure 4A), which coincides with other studies (Schulte et al., 1987; Carafa et al., 2005), but fibers are quite abundant in cortical tissues. Cortical fibers have a secondary cell wall that differs from secondary cell wall in the xylem. The major polysaccharide in stele tracheids is β-(1,4)-xylan, while labeling by the mannan-recognizing antibody LM21 was only poorly detectable (Figure 5). In cortical fibers, the secondary cell wall was heavily labeled with LM21 with an intensity similar to the primary cell wall. Comparison of sugar composition in various stem orders (Figure 3) demonstrated that the proportion of mannose among cell wall-derived monomers remained high after cell wall thickening in fibers, indicating that their secondary cell walls, similar to primary cell walls, were enriched in mannan. Mannans were also detected in cell walls of sclerified and collenchymatous tissues of ferns and *Equisetum* (Leroux et al., 2015), but the definite cell types were not identified. The abundant thick-walled cells in the cortex of *P. nudum* are typical sclerenchyma fibers (Figure 3). The significant cell length, together with tapered cell ends, indicate the pronounced intrusive growth and suggests *P. nudum* as an example of early vascular land plants to study the evolution of this process important for fiber differentiation.

In cortical fibers, binding of LM11 that recognizes β-(1,4)-xylan was specific only for the secondary cell wall, the same as in tracheids, but in the fibers it occurred less evenly and was barely detected in some fiber cells (Figures 5E–J). Deposition of lignin and xylan in *P. nudum* fibers goes in parallel to each other (Figure 7) indicating the possibility of regulation by the same master-switch transcription factors, similarly to the situation in higher plants (Mitsuda et al., 2007; Zhong et al., 2007). However, in elder stem order, staining with phloroglucinol-HCl spreads in fibers over all cell wall layers, while binding of LM11 is restricted to secondary cell walls (Figure 7). Another difference between secondary cell walls in tracheids and cortical fibers of *P*. *nudum* is the presence of an innermost cell wall layer that binds the set of antibodies – LM15, LM5 and LM6 specific, respectively, for xyloglucan, α-(1,5)-arabinan and β-(1,4)-galactan– that do not label the major layer of thickened cell wall (Figures 5I–L, 6A–F, Table 1). Such a layer is absent in *P*. *nudum* tracheids.

Thus, the composition and structure of secondary cell walls in *P*. *nudum* is not uniform in different tissues. Cortical fibers have both mannan and β-(1,4)-xylan in their secondary cell walls, while in tracheids, the matrix of secondary cell wall is largely xylan-based. This may be coupled to the degree of lignification, since staining with phloroglucinol-HCl was much more pronounced and more uniform in stele cells with xylan-based thickened cell walls, than in cortical fibers (Figures 3A,B). In early land plants of major taxonomic groups, β-(1,4)-D-xylans, if found, are detected immunochemically only in secondary cell walls (Carafa et al., 2005), while mannan can be found both in primary and secondary cell walls, as is evident from the study of cortical fibers in *P*. *nudum*.

The thickened cell walls in characterized cortical fibers of *P. nudum* stems clearly differ from the widely described fibers of angiosperm plants in proportion of mannans. In higher plant fibers, mannose-containing polymers comprise 2–5%, both in secondary (Ha et al., 2002; Donev et al., 2018) and tertiary (Rihouey et al., 2017) cell walls, while the major matrix polysaccharide is xylan, which proportion accounts for 30–40% (Smith et al., 2017; Donev et al., 2018). In cortical fibers of *P. nudum* the situation is the opposite (Figures 3, 5, 7) and this is the first described example of sclerenchyma fibers with mannan-based cell wall matrix.

Additional early vascular plants, including the other species of the *Psilotaceae*, should be studied in order to test the possibility that the composition of cell wall polymers in this group represents an evolutionary pattern, or reflects specific adaptations of individual species. Further research to relate the diversity of secondary cell wall composition to the differences of cell wall properties and to their function in various tissues of the plant body would enable us to understand the reasons and the consequences of plant cell wall evolution.

## Data Availability Statement

All datasets generated for this study are included in the article/Supplementary material.

## Author Contributions

TC and MA performed the microscopy and immunochemistry. PM carried out the HPAEC-PAD method development. OT prepared the AIR and made the hydrolysis. LK analyzed the monosaccharide composition. TC, SL-Y, and TG wrote of the manuscript. All co-authors have reviewed and approved the manuscript.

## Conflict of Interest

The authors declare that the research was conducted in the absence of any commercial or financial relationships that could be construed as a potential conflict of interest.

## References

[B1] Blake A. W. Marcus S. E. Copeland J. E. Blackburn R. S. Knox J. P. (2008). In situ analysis of cell wall polymers associated with phloem fibre cells in stems of hemp, *Cannabis sativa* L. *Planta* 228 1–13. 10.1007/s00425-008-0713-5 18299887

[B2] Bowling A. J. Vaughn K. C. (2008). Immunocytochemical characterization of tension wood: gelatinous fibers contain more than just cellulose. *Am. J. Bot.* 95 655–663. 10.3732/ajb.2007368 21632390

[B3] Boyce C. K. Cody G. D. Fogel M. L. Hazen R. M. Alexander C. M. O. Knoll A. H. (2003). Chemical evidence for cell wall lignification and the evolution of tracheids in early Devonian plants. *Intern. J. Plant Sci.* 164 691–702. 10.1086/377113

[B4] Boyce C. K. Zwieniecki M. A. Cody G. D. Jacobsen C. Wirick S. Knoll A. H. (2004). Evolution of xylem lignification and hydrogel transport regulation. *Proc. Natl. Acad. Sci. U.S.A.* 101 17555–17558. 10.1073/pnas.0408024101 15574502 PMC536047

[B5] Carafa A. Duckett J. G. Knox J. P. Ligrone R. (2005). Distribution of cell-wall xylans in bryophytes and tracheophytes: new insights into basal interrelationships of land plants. *New Phytol.* 168 231–240. 10.1111/j.1469-8137.2005.01483.x 16159336

[B6] Chavan R. R. Fahey L. M. Harris P. J. (2015). Quantification of (1→4)-ß-d-galactans in compression wood using an immuno-dot assay. *Plants* 4 29–43. 10.3390/plants4010029 27135316 PMC4844332

[B7] Donev E. Gandla M. L. Jönsson L. J. Mellerowicz E. (2018). Engineering non-cellulosic polysaccharides of wood for the biorefinery. *Front. Plant Sci.* 9:1537. 10.3389/fpls.2018.01537 30405672 PMC6206411

[B8] Fahn A. (1990). *Plant Anatomy*, 4th Edn, Oxford: Pergamon Press.

[B9] Ford S. O. (1904). The anatomy of *Psilotum triquetrum*. *Ann. Bot.* 18 589–608.

[B10] Friedman W. E. Cook M. E. (2000). The origin and early evolution of tracheids in vascular plants: integration of palaeobotanical and neobotanical data. *Philos. Trans. R. Soc. Lond B* 355 857–868. 10.1098/rstb.2000.0620 10905614 PMC1692781

[B11] Gierlinger N. Sapei L. Paris O. (2008). Insights into the chemical composition of *Equisetum hyemale* by high resolution Raman imaging. *Planta* 227 969–980. 10.1007/s00425-007-0671-3 18057960 PMC2756348

[B12] Gifford E. M. Foster A. S. (1989). *Morphology and Evolution of Vascular Plants*, 3rd Edn, New York: W. H. Freeman and Company.

[B13] Gorshkova T. Brutch N. Chabbert B. Deyholos M. Hayashi T. Lev-Yadun S. (2012). Plant fiber formation: state of the art, recent and expected progress, and open questions. *Crit. Rev. Plant Sci.* 31 201–228. 10.1080/07352689.2011.616096

[B14] Gorshkova T. Chernova T. Mokshina N. Ageeva M. Mikshina P. (2018). Plant ‘muscles’: fibers with a tertiary cell wall. *New Phytol.* 218 66–72. 10.1111/nph.14997 29364532

[B15] Guedes F. T. P. Laurans F. Quemener B. Assor C. Lainé-Prade V. Boizot N. (2017). Non-cellulosic polysaccharide distribution during G-layer formation in poplar tension wood fibers: abundance of rhamnogalacturonan I and arabinogalactan proteins but no evidence of xyloglucan. *Planta* 246 857–878. 10.1007/s00425-017-2737-1 28699115

[B16] Ha M.-A. MackKinnon I. M. Šturcová A. Apperley D. C. McCann M. C. Turner S. R. (2002). Structure of cellulose-deficient secondary cell walls from the irx3 mutant of *Arabidopsis thaliana*. *Phytochemistry* 61 7–14. 10.1016/S0031-9422(02)00199-1 12165296

[B17] Harris P. J. (2005). “Diversity in plant cell walls,” in *Plant Diversity And Evolution: Genotypic And Phenotypic Variation In Higher Plants*, ed. Henry R. J. (Wallingford: CAB International Publishing), 201–227. 10.1079/9780851999043.0201

[B18] Hill A. F. (1952). *Economic Botany. A Textbook of Useful Plants and Plant Products.* New York, NY: McGraw Hill Book Co., Inc.

[B19] Hsieh Y. S. Y. Harris P. J. (2019). Xylans of red and green algae: what is known about their structures and how they are synthesised? *Polymers* 11 354. 10.3390/polym11020354 30960338 PMC6419167

[B20] Jensen W. A. (1962). *Botanical Histochemistry: Principles and Practice.* San Francisco: W. H. Freeman and Company.

[B21] Jones L. Seymour G. B. Knox J. P. (1997). Localization of pectic galactan in tomato cell walls using a monoclonal antibody specific to (1[→]4)-[beta]-d-galactan. *Plant Physiol.* 113 1405–1412. 10.1104/pp.113.4.1405 12223681 PMC158264

[B22] Kenrick P. Crane P. R. (1997). *The Origin and Early Diversification of Land Plants: A Cladistic Study.* Washington, DC: Smithsonian Institution Press.

[B23] Kim J. S. Daniel G. (2012). Immunolocalization of hemicelluloses in *Arabidopsis thaliana* stem. Part I: temporal and spatial distribution of xylans. *Planta* 236 1275–1288. 10.1007/s00425-012-1686-y 22711286

[B24] Knox J. P. Linstead P. J King J. Cooper C. Roberts K. (1990). Pectin esterification is spatially regulated both within cell walls and between developing tissues of root apices. *Planta* 181 512–521. 10.1007/BF00193004 24196931

[B25] Knox J. P. Linstead P. J. Peart J. Cooper C. Roberts K. (1991) Developmentally regulated epitopes of cell surface arabinogalactan proteins and their relation to root tissue pattern formation. *Plant J.* 1 317–326. 10.1046/j.1365-313X.1991.t01-9-00999.x 21736649

[B26] Kulkarni A. R. Peña M. J. Avci U. Mazumder K. Urbanowicz B. R. Pattathil S. (2012). The ability of land plants to synthesize glucuronoxylans predates the evolution of tracheophytes. *Glycobiology* 22 439–451. 10.1093/glycob/cwr117 22048859

[B27] Leroux O. Bagniewska-Zadworna A. Rambe S. K. Knox J. P. Marcus S. E. Bellefroid E. (2011). Non-lignified helical cell wall thickenings in root cortical cells of Aspleniaceae (Polypodiales): histology and taxonomical significance. *Ann. Bot.* 107 195–207. 10.1093/aob/mcq225 21118842 PMC3025727

[B28] Leroux O. Sørensen I. Marcus S. E. Viane R. L. Willats W. G. Knox J. P. (2015). Antibody-based screening of cell wall matrix glycans in ferns reveals taxon, tissue and cell-type specific distribution patterns. *BMC Plant Biol.* 15 56. 10.1186/s12870-014-0362-8 25848828 PMC4351822

[B29] Ligrone R. Vaughn K. C. Renzaglia K. S. Knox J. P. Duckett J. G. (2002). Diversity in the distribution of polysaccharide and glycoprotein epitopes in the cell walls of bryophytes: new evidence for multiple evolution of water-conducting cells. *New Phytol.* 156 491–508. 10.1046/j.1469-8137.2002.00538.x 33873570

[B30] Marcus S. E. Blake A. W. Benians T. A. Lee K. J. Poyser C. Donaldson L. (2010). Restricted access of proteins to mannan polysaccharides in intact plant cell walls. *Plant J.* 64 191–203. 10.1111/j.1365-313X.2010.04319.x 20659281

[B31] Marcus S. E. Verhertbruggen Y. Hervé C. Ordaz-Ortiz J. J. Farkas V. Pedersen H. L. (2008). Pectic homogalacturonan masks abundant sets of xyloglucan epitopes in plant cell walls. *BMC Plant Biol.* 8:60. 10.1186/1471-2229-8-60 18498625 PMC2409341

[B32] McCartney L. Marcus S. E. Knox J. P. (2005). Monoclonal antibodies to plant cell wall xylans and arabinoxylans. *J. Histochem. Cytochem.* 53 543–546. 10.1369/jhc.4B6578.2005 15805428

[B33] McDougall G. J. Morrison I. M. Stewart D. Weyers J. D. B. Hillman J. R. (1993). Plant fibres: botany, chemistry and processing for industrial use. *J. Sci. Food Agric.* 62 1–20. 10.1002/jsfa.2740620102

[B34] Meikle P. J. Hoogenraad N. J. Bonig I. Clarke A. E. Stone B. A. (1994). A (1→3, 1→4)-β-glucan-specific monoclonal antibody and its use in the quantitation and immunocytochemical location of (1→3, 1→4)-β-glucans. *Plant J.* 5 1–9. 10.1046/j.1365-313X.1994.5010001.x 8130794

[B35] Mellerowicz E. J. Baucher M. Sundberg B. Boerjan W. (2001). Unravelling cell wall formation in the woody dicot stem. *Plant Mol. Biol.* 47 239–274. 10.1023/A:1010699919325 11554475

[B36] Merced A. Renzaglia K. (2014). Developmental changes in guard cell wall structure and pectin composition in the moss *Funaria*: implications for function and evolution of stomata. *Ann. Bot.* 114 1001–1010. 10.1093/aob/mcu165 25129633 PMC4171074

[B37] Merced A. Renzaglia K. (2019). Contrasting pectin polymers in guard cell walls of *Arabidopsis* and the hornwort *Phaeoceros reflect* physiological differences. *Ann. Bot.* 123 579–585. 10.1093/aob/mcy168 30202908 PMC6417473

[B38] Mitsuda N. Iwase A. Yamamoto H. Yoshida M. Seki M. Shinozaki K. (2007). NAC transcription factors, NST1 and NST3, are key regulators of the formation of secondary walls in woody tissues of Arabidopsis. *Plant Cell* 19 270–280. 10.1105/tpc.106.047043 17237351 PMC1820955

[B39] Popper Z. A. Fry S. C. (2003). Primary cell wall composition of bryophytes and charophytes. *Ann. Bot.* 91 1–12. 10.1093/aob/mcg013 12495914 PMC4240358

[B40] Popper Z. A. Fry S. C. (2004). Primary cell wall composition of pteridophytes and spermatophytes. *New Phytol.* 164 165–174. 10.1111/j.1469-8137.2004.01146.x 33873476

[B41] Rihouey C. Paynel F. Gorshkova T. Morvan C. (2017). Flax fibers: assessing the non-cellulosic polysaccharides and an approach to supramolecular design of the cell wall. *Cellulose* 24 1985–2001. 10.1007/s10570-017-1246-5

[B42] Ruhfel B. R. Gitzendanner M. A. Soltis P. S. Soltis D. E. Burleigh J. G. (2014). From algae to angiosperms-inferring the phylogeny of green plants (*Viridiplantae*) from 360 plastid genomes. *BMC Evol. Biol.* 14 23. 10.1186/1471-2148-14-23 24533922 PMC3933183

[B43] Schulte P. J. Gibson A. C. Nobel P. S. (1987). Xylem anatomy and hydraulic conductance of *Psilotum nudum*. *Am. J. Bot.* 74 1438–1445. 10.2307/2444320

[B44] Silva G. B. Ionashiro M. Carrara T. B. Crivellari A. C. Tiné M. A. Prado J. (2011). Cell wall polysaccharides from fern leaves: evidence for a mannan-rich Type III cell wall in *Adiantum raddianum*. *Phytochemistry* 72 2352–2360. 10.1016/j.phytochem.2011.08.020 21955619

[B45] Smith P. J. Wang H. York W. S. Pena M. J. Urbanowicz B. R. (2017). Designer biomass for next-generation biorefineries: leveraging recent insights into xylan structure and biosynthesis. *Biotechnol. Biof.* 10 286. 10.1186/s13068-017-0973-z PMC5708106 29213325

[B46] Sørensen I. Pettolino F. A. Bacic A. Ralph J. Lu F. O’Neill M. A. (2011). The charophycean green algae provide insights into the early origins of plant cell walls. *Plant J.* 68 201–211. 10.1111/j.1365-313X.2011.04686.x 21707800

[B47] Sorieul M. Dickson A. Hill S. J. Pearson H. (2016). Plant fibre: molecular structure and biomechanical properties, of a complex living material, influencing its deconstruction towards a biobased composite. *Materials* 9 E618. 10.3390/ma9080618 28773739 PMC5509024

[B48] Vahdati F. B. Mehrvarz S. S. Naqinezhad A. Shavvon R. S. (2014). The morphological and anatomical reinvestigation of the *Psilotum nudum*, in Hyrcanian forests, N Iran. *Taxon. Biosystemat.* 6 87–96.

[B49] Valavan R. E. Mayilsamy M. Rajendran A. (2016). Psilotum nudum: a new medicinal pteridophyte record for the cryptogamic flora of sirumalai hills, Dindigul district, Tamilnadu, India. *Shanlax Intern. J. Arts Sci. Hum.* 3 48–52.

[B50] Viana A. G. Noseda M. D. Gonçalves A. G. Duarte M. E. Yokoya N. Matulewicz M. C. (2011). ß-D-(1–>4), ß-D-(1→3) ‘mixed linkage’ xylans from red seaweeds of the order Nemaliales and Palmariales. *Carbohydrate Res.* 346 1023–1028. 10.1016/j.carres.2011.03.013 21507387

[B51] Willats W. G. Marcus S. E. Knox J. P. (1998). Generation of a monoclonal antibody specific to (1→5)-α-L-arabinan. *Carbohydr. Res.* 308 149–152. 10.1016/S0008-6215(98)00070-6 9675359

[B52] Zhong R. Richardson E. A. Ye Z. H. (2007). Two NAC domain transcription factors, SND1 and NST1, function redundantly in regulation of secondary wall synthesis in fibers of *Arabidopsis*. *Planta* 225 1603–1611. 10.1007/s00425-007-0498-y 17333250

